# NLRC3 attenuates osteoclastogenesis by limiting TNFα^+^ Th17 cell response in osteoporosis

**DOI:** 10.1007/s00109-024-02422-y

**Published:** 2024-03-04

**Authors:** Lingyan Ren, Guangjun Liu, Yun Bai, Liling Gu, Yuan Wang, Li Sun

**Affiliations:** 1 Department of Orthopedics, Guizhou Provincial People’s Hospital, Guiyang, Guizhou Province, 550003 China; 2 Antenatal Diagnosis Centre, Guizhou Provincial People’s Hospital, Guiyang, Guizhou Province, 550003 China; 3 Institute of Traumatic Orthopedics, The 80th, Army Hospital of the Chinese People’s Liberation Army, Weifang Shandong Province, 500000 China; 4 Department of Rehabilitation, Guizhou Provincial People’s Hospital, Guiyang, Guizhou Province, 550003 China; 5 Department of Orthopedics, TongRen Hospital, School of Medicine Shanghai, Jiao Tong University, Shanghai, 200336 China

**Keywords:** NOD-like receptor (NLR) family CARD domain containing 3 (NLRC3), Osteoporosis, Osteoclast, Th17 cell, Bone remodeling

## Abstract

**Abstract:**

NOD-like receptor family CARD domain containing 3 (NLRC3) is the intracellular protein belonging to NLR (NOD-like receptor) family. NLRC3 can negatively regulate inflammatory signal transduction pathways within the adaptive and innate immunocytes. However, studies need to elucidate the biological role of NLRC3 in bone remodeling. Herein, our study proved that NLRC3 prevents bone loss by inhibiting TNFα^+^ Th17 cell responses. In osteoporosis, NLRC3 attenuated TNFα^+^ Th17 cell accumulation in the bone marrow. However, osteoporosis (OP) development was aggravated without affecting bone marrow macrophage (BMM) osteoclastogenesis in NLRC3-deficient ovariectomized (OVX) mice. In this study, we transferred the wild-type and NLRC3^−/−^ CD4^+^ cells into Rag1^−/−^ mice. Consequently, we evidenced the effects of NLRC3 in CD4^+^ T cells on inhibiting the accumulation of TNFα + Th17 cells, thus restricting bone loss in the OVX mice. Simultaneously, NLRC3^−/−^ CD4^+^ T cells promoted the recruitment of osteoclast precursors and inflammatory monocytes into the OVX mouse bone marrow. Mechanism-wise, NLRC3 reduced the secretion of TNFα + Th17 cells of RANKL, MIP1α, and MCP1, depending on the T cells. In addition, NLRC3 negatively regulated the Th17 osteoclastogenesis promoting functions via limiting the NF-κB activation. Collectively, this study appreciated the effect of NLRC3 on modulating bone mass via adaptive immunity depending on CD4^+^ cells. According to findings of this study, NLRC3 may be the candidate anti-OP therapeutic target.

**Key messages:**

- NLRC3 negatively regulated the Th17 osteoclastogenesis promoting functions via limiting the NF-κB activation.
- NLRC3 may be the candidate anti-OP therapeutic target.

**Supplementary Information:**

The online version contains supplementary material available at 10.1007/s00109-024-02422-y.

## Introduction

Osteoporosis (OP) is a widespread and serious metabolic bone disorder, characterized by a decrease in bone volume, quality, and mineral density [1–3]. Excessive bone resorption by osteoclast (OC) will result in low bone mass, which manifests the signs of osteoporosis. Therefore, OC hyperactivation is commonly accepted as the chief pathogenesis of osteoporosis. OCs originate from monocytic precursors in the bone marrow, and M-CSF is indispensable for OC differentiation and sustenance [4]. Studies have shown that enhanced OC activity is related to the accumulation of CD11b^hi^Ly6C^hi^ inflammatory monocytes and CD11b^−/l^° Ly6C^hi^ cells that have been described as OC precursors under pathological conditions [5]. As demonstrated by recent studies, the aberrant OC activation interacts with and is regulated by immune cells, especially T cells [6–8]. However, studies need to reveal further the mechanism by which T cells are activated and promote osteoclastogenesis.

Bone destruction associated with chronic inflammation has been attributed to the activated CD4^+^ T cells. For the mice and human inflammatory bone destruction, studies have evidenced that TNFα+ Th17 T cells are effective inducers of OC-differentiation [9]. According to the existing studies, Th17 cells together with the corresponding marker cytokine IL-17A play important roles in bone destruction in periodontitis through enhancing OC formation [10, 11]. TNF induces osteoclastogenesis by promoting OC-precursor cell proliferation or activating the differentiated OCs through the RANK/RANKL signaling pathway.

NLRC3, which belongs to NLR family, was initially discovered from T cells, whose expression has been mentioned in diverse immune cells and tissues [12–14]. NLRC3 mitigates T cell growth and activation, as well as cytokine generation in CD4^+^ T cells. Although the functions and effects of NLRC3 on immune cells have been extensively studied, the role and mechanisms related to its function in OC activation and bone remodeling are still unclear [12].

This study investigated the roles and mechanisms by which NLRC3 regulated Th17 cell activation and osteoclastogenesis during OP. This study has evidence to satisfactorily reveal that NLRC3 attenuates the osteoclastogenesis promoting CD4^+^ T cells’ function and capacity of recruiting CD11b^−/l^°Ly6C^hi^ inflammatory monocytes into the bone marrow, thus restricting OP development. NLRC3 negatively regulated Th17 cells in vivo and in vitro through NK-κB pathway. Our findings indicated that NLRC3 was the candidate anti-OP therapeutic target to manage the dysregulated adaptive immunity.

## Results

### NLRC3-deficiency aggravates osteoporosis development in OVX mice without affecting BMM osteoclastogenesis

**Fig. 1:**
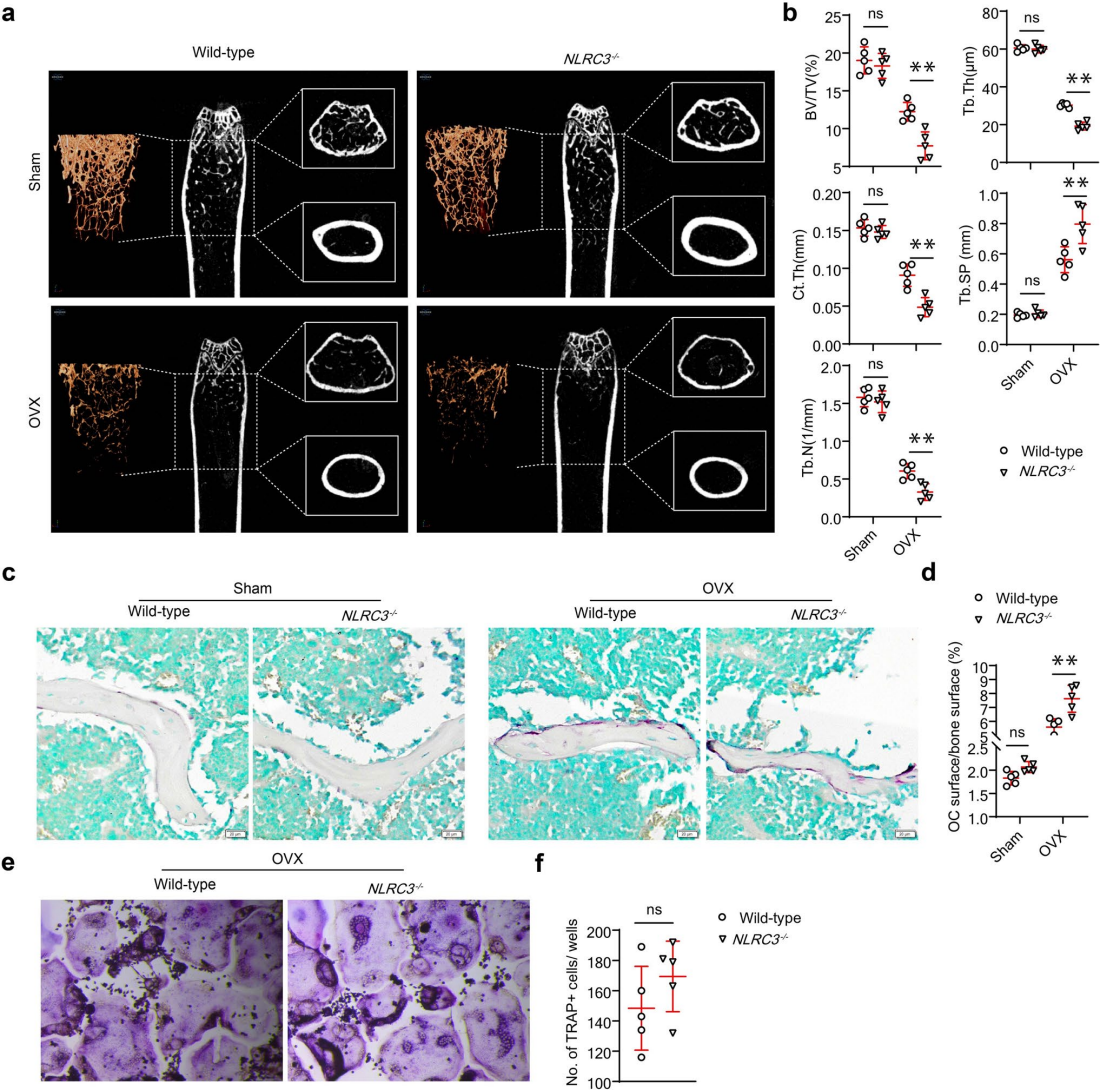
NLRC3-deficiency aggravates osteoporosis development in OVX mice without affecting BMM osteoclastogenesis. **a** Representative 3D micro-computer tomography (μCT) images of distal femur. **b** Quantitative μCT analysis of BV/TV, Tb. N, Tb. Th, Ct. Th, and Tb. Sp. **c** TRAP staining for femurs sections of each group. Scale bar, 20 μm. **d** Ratio of OC surface to bone surface analyzed quantitatively. **e**, **f** BMMs obtained in WT or NLRC3^−/−^ mice were subjected to 7 days of culture with 20 ng/mL M-CSF and 50 ng/mL RANKL. **e** Representative images of TRAP staining. **f** Quantitative analysis of TRAP staining. Scale bars = 200 μm. Results are presented in a form of mean ± SD. *P < 0.05, **P < 0.01

As demonstrated, NLRC3 attenuated CD4^+^ T cell autoimmunity and responses within dendritic cells (DCs); also, it negatively regulated T cells and macrophages [12–15]. Meanwhile, the activated T lymphocytes were the primary source of RANKL that had been the core molecule in osteoclastogenesis. However, studies have not adequately elucidated the specific role of NRLC3 in osteoclastogenesis. To overcome this shortcoming, we investigated how NLRC3 affected OC formation. Therefore, this study evaluated NLRC3’s function in the osteoporotic in vivo OVX mice model. To begin with the study, NLRC3^−/−^ mice were generated and implemented with osteoporotic OVX. Next, the µCT analysis showed that NLRC3^−/−^ OVX mice displayed a lower bone mass than OVX mice (Fig. 1a). Furthermore, quantification analysis showed that, in OVX mice, NLRC3-deficiency evidently reduced trabecular number (Tb. N), trabecular bone volume fraction (BV/TV), trabecular separation increase (Tb. Sp), trabecular bone thickness (Tb. Th), and cortical bone thickness (Ct. Th), with statistical significance (*p* < 0.01) (Fig. 1b). As revealed by TRAP-stained distal femurs, NLRC3^−/−^ OVX mice had remarkably increased ratio of OC surface to bone surface compared with OVX mice; moreover, no significance was detected between the sham and NLRC3^−/−^ groups (Fig. 1c, d). Subsequently, to identify NLRC3’s direct function in OC differentiation, BMMs were cultured from NLRC3^−/−^ OVX mice or wild-type OVX mice. TRAP stain showed no significance between the NLRC3^−/−^ group and wild-type control (Fig. 1e, f). The above observations indicate that NLRC3-deficiency aggravates osteoporosis development in OVX mice without directly affecting osteoclastogenesis in BMM.

### Accumulation of TNF-α^+^ Th17 cells in OVX-treated NLRC3^−/−^ mouse bone marrow

**Fig. 2:**
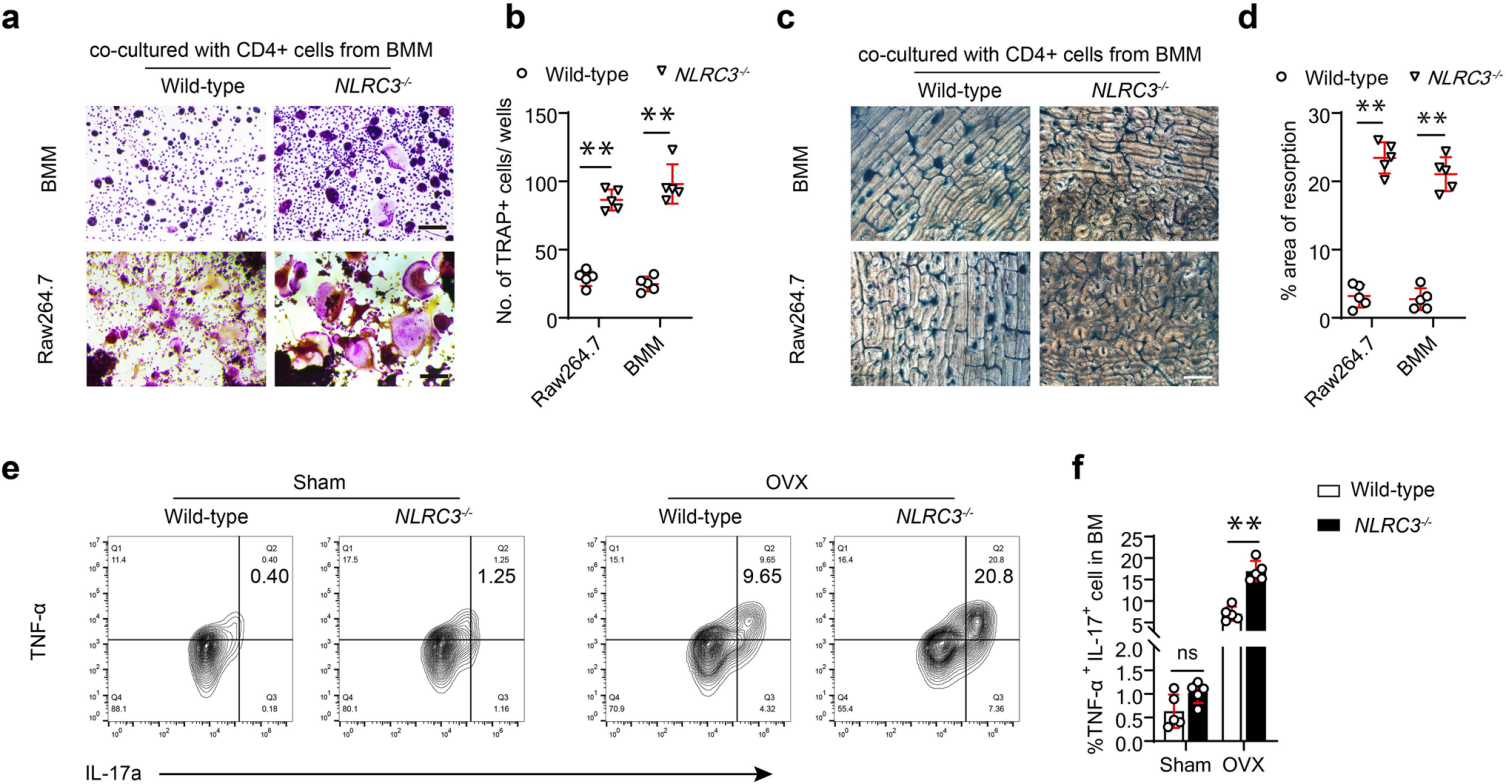
Accumulation of TNF-α^+^ Th17 cells representing an osteoclastogenic T cell subset into the bone marrow of NLRC3^−/−^ mice with OVX. **a**, **b** Sorted CD4^+^ cells obtained in WT or NLRC3^−/−^ mice bone marrow were subjected to 7 days of culture with wild-type monocytes or Raw264.7 cells using 20 ng/mL M-CSF and 50 ng/mL RANKL. **a** Representative images of TRAP staining for BMM cells. **b** TRAP staining analyzed quantitatively. Scale bars, 200 μm. **c** Typical images showing bovine bone section-based resorption assay. **d** Bone resorption area quantified using bone sections. Scale bar, 200 μm. **e** IL-17A and TNF-α expression in bone marrow CD4^+^ T cells obtained in WT or NLRC3^−/−^ mice after OVX or sham surgery was assessed by intracellular flow cytometry. **f** Quantification of **e**. Results were shown in a form of mean ± SD. *P < 0.05, **P < 0.01

Studies indicate that bone marrow TNFα-producing Th17 cells serve as the inducers of osteoclastogenesis. Since NLRC3 rarely had any direct effect on the in vitro BMM osteoclastogenesis, we conducted further analysis for testing bone marrow CD4^+^ T cells’ ability of inducing osteoclastogenesis. Therefore, we separated CD4^+^ T cells from NLRC3^**−/−**^ OVX or WT mouse bone marrow, and their osteoclastogenic effect was tested in an in vitro co-culture with wild-type BMMs (OC precursors), as well as in co-culture with the mouse macrophage cell line RAW264.7. The TRAP stain results show that BMMs from NLRC3^−/−^ OVX mice exhibited the more active osteoclastogenesis (Fig. 2a, b). Moreover, pit formation assay on both osteo surfaces showed significantly increased bone resorption activity of osteoclast in NLRC3^−/−^ OVX mice (Fig. 2c, d).

Among CD4^+^ T cells, the Th17 TNFα+ T cells proved to be the potent inducer of OC differentiation in the context of inflammation. Furthermore, bone marrows were isolated from the NLRC3^−/−^ or wild-type OVX mice to investigate the frequency of TNFα-producing Th17 cells in NLRC3^−/−^ mice with OVX. Flow cytometry results showed NLRC3 deficiency to cause more TNFα-producing Th17 cell accumulation in the bone marrow (Fig. 2e, f). These results indicate that the CD4^+^ T cell population might increase osteoclastogenesis in the NLRC3^−/−^ OVX mice. Altogether, these results confirmed that TNF-α^+^ Th17 cells are related to the osteoclastogenesis of NLRC3^−/−^ OVX mouse bone marrow.

### NLRC3 suppresses the accumulation of TNFα^+^ Th17 cells within CD4^+^ T cells and restricts bone loss of OVX mice

**Fig. 3:**
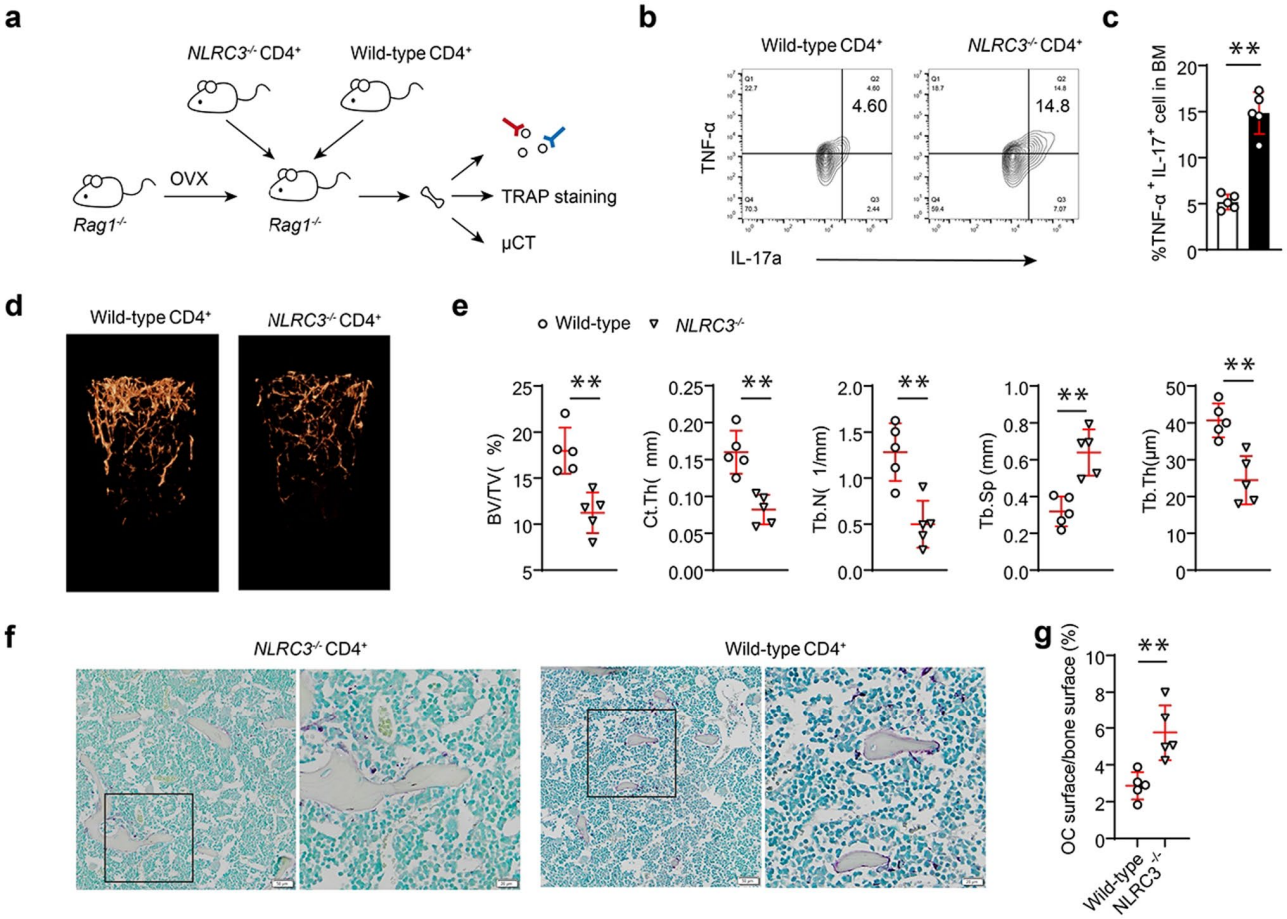
NLRC3 inhibits the accumulation of TNFα^+^ Th17 cells in CD4^+^ T cells and restricts bone loss in ovariectomized mice. **a** Sketch map showing CD4^+^ adaptive transfers of WT wild-type or NLRC3^−/−^ cells into Rag1^−/−^ mice. **b** IL-17A and TNF-α expression in bone marrow CD4^+^ T cells obtained in WT or NLRC3^−/−^ CD4^+^ cell-transfected Rag1^−/−^ mice were analyzed through intracellular flow cytometry. **c** Quantification of TNF-α Th17 cell frequency in **b**. **d** Typical 3DμCT images showing distal femur in wild-type or NLRC3^−/−^ CD4^+^ cell-transfected Rag1^−/−^ mice. **e** Quantitative μCT assay on trabecular BV/TV, Ct. Th, Tb. N, Tb. Th, and Tb. Sp. **f** TRAP staining of femurs sections of each group. Scale bar, 20 μm. **g** Ratio of OC surface to bone surface analyzed quantitatively. Results were presented in a form of mean ± SD. *P < 0.05, **P < 0.01

Thus far, our observations suggest that TNF-α + Th17 cells accumulate within the NLRC3^−/−^ OVX mouse bone marrow; however, the effects of NLRC3 on promoting OC differentiation within CD4^+^ T cells is still unknown. Therefore, for elucidating NLRC3’s specific effect on CD4^+^ T cells, the CD4^+^ T cell adoptive transfers were conducted, with the co-transfection of NLRC3^−/−^ or WT CD4^+^ T cells in Rag1^−/−^ OVX mice (Fig. 3a). The results showed a significant increase of TNF-α^+^Th17 cells in Rag1^−/−^ OVX mice transferred with NLRC3^−/−^ compared with WT CD4^+^ T cells in controls (Fig. 3b, c). Consequently, µCT scanned images of the dissected femurs showed a lower bone mass in NLRC3^−/−^CD4^+^ T cell-transfected Rag1^−/−^ OVX mice than WT CD4^+^ T cell-transfected counterparts (Fig. 3d, e). TRAP staining also showed significantly increased the ratio of OC surface to bone surface of NLRC3^−/−^ CD4^+^ T cell-transfected OVX mice (Fig. 3f, g). The above results indicate that NLRC3 expression in CD4^+^ T cells inhibits TNFα+ Th17 cell accumulation, thus restricting bone loss in OVX mice.

### CD4^+^T cells obtained in NLRC3-deficient mice promote the recruitment of OC precursors into the bone marrow in osteoporosis

**Fig. 4:**
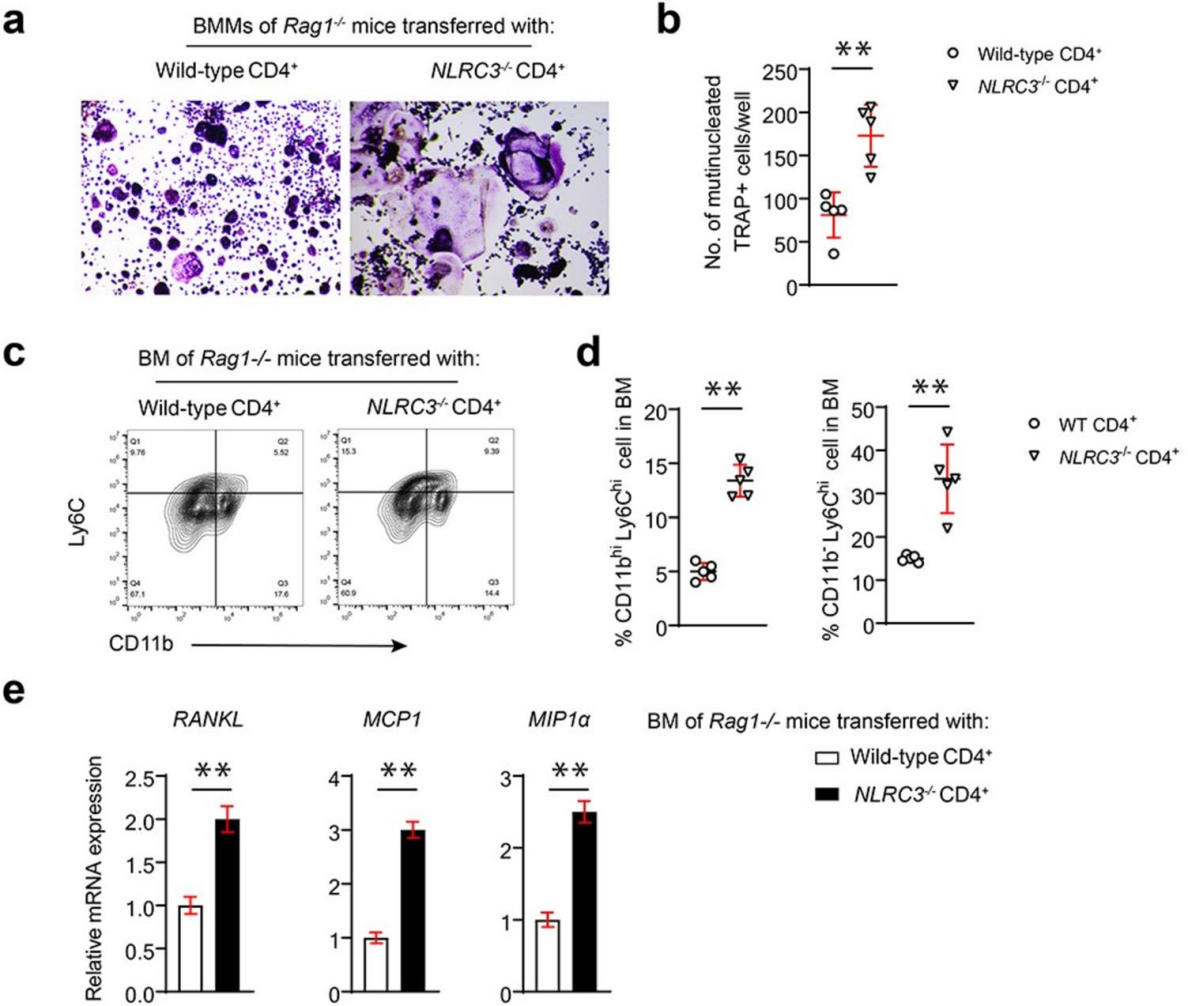
CD4^+^ T cells obtained in NLRC3-deficient mice promote the recruitment of OC precursors into the bone marrow in osteoporosis. **a**, **b** BMMs from NLRC3^−/−^ CD4^+^ or WT cell-transfected Rag1^−/−^ mice were subjected to 7 days of culture with 20 ng/mL M-CSF and 50 ng/mL RANKL. **a** Representative TRAP staining images. **b** TRAP staining analyzed quantitatively. Scale bars = 200 μm. **c** Typical flow cytometric analysis of CD11b^hi^Ly6C^hi^ and CD11b^−^Ly6C^hi^ cells from bone marrow cells of NLRC3^−/−^ or WT CD4^+^ cell-transfected Rag1^−/−^ mice. **d** Quantification of the frequency of CD11b^hi^Ly6C^hi^ and CD11b^−^Ly6C^hi^ cells in **c**. **e** The relative mRNA expression in bone marrow cells of WT or NLRC3^−/−^ CD4^+^ cell-transfected Rag1^−/−^ mice. Results were presented in a form of mean ± SD. *P < 0.05, **P < 0.01

Aggravated osteoclastogenesis leads to bone destruction in OVX mice due to the accumulation of more OC precursors into the bone marrow. Thus, OC differentiation was performed in vitro equal bone marrow cell numbers obtained in Rag1^−/−^ mice with transferred WT and NLRC3^−/−^ CD4^+^ T cells. TRAP staining demonstrated an increase in osteoclastogenesis only in bone marrow cells from Rag1^−/−^ mice transfected with NLRC3^−/−^ CD4^+^ T cells, which is consistent with the bone loss observed in these animals (Fig. 4a, b). Moreover, flow cytometric analysis showed increased CD11b^hi^Ly6C^hi^ inflammatory monocytes and CD11b^−/l^° Ly6C^hi^ cells as the precursors of OC from bone marrow in NLRC3^−/−^ CD4^+^ T cell-transfected Rag1^−/−^ mice compared with WT CD4^+^ T cell-transfected counterparts (Fig. 4c, d). Next, this study analyzed the chemokine levels that attract inflammatory monocytes, such as MCP1, MIP1α, and RANKL (Fig. 4e). The chemokine expression of the bone marrow cells in NLRC3^−/−^ CD4^+^ T cell-transfected Rag1^−/−^ mice increased compared with the bone marrow from CD4^+^ T cell-transfected counterparts. Altogether, the above findings reveal that NLRC3 within CD4^+^ T cells attenuated recruitment of CD11b^hi^Ly6C^hi^ inflammatory monocytes and CD11b^–/l^° Ly6C^hi^ cells as OC precursors into the bone marrow by inhibiting the expression of MCP1, MIP1α, and RANKL in OVX mice.

### NLRC3-deficient mice are more susceptible to inflammatory bone loss

**Fig. 5:**
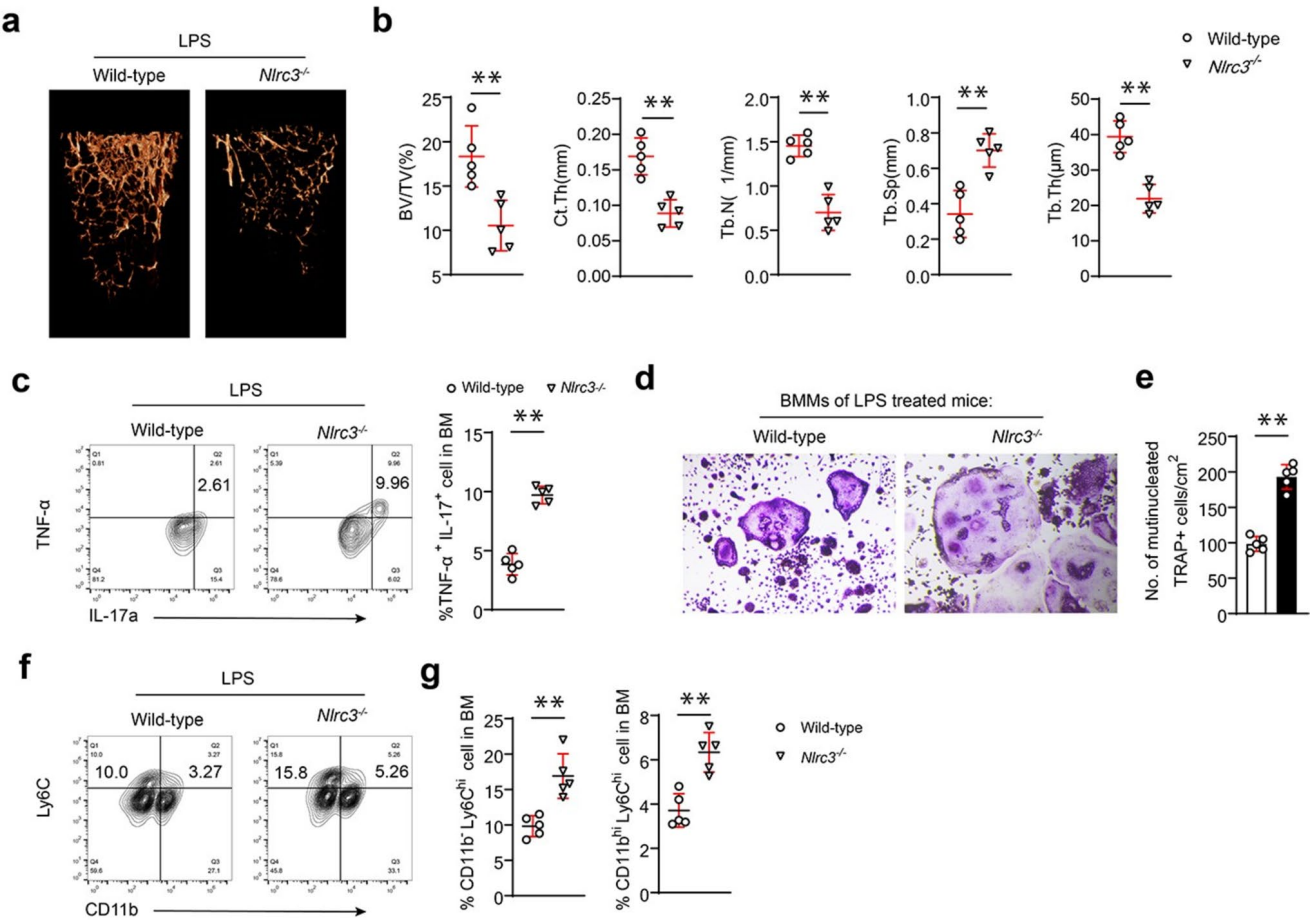
NLRC3-deficient mice are more susceptible to inflammatory bone loss. **a** Typical 3DμCT images showing distal femur in the WT or NLRC3^−/−^ mice subjected to lipopolysaccharide (LPS). **b** Quantitative μCT analysis on BV/TV, Ct. Th, Tb. N, Tb. Th, and Tb. Sp. **c** IL-17A and TNF-α expression in bone marrow CD4^+^ T cells obtained in LPS-treated WT or NLRC3^−/−^ mice were assessed by intracellular flow cytometry. **d**, **e** BMMs from LPS-treated NLRC3^−/−^ or WT mice were subjected to 7 days of culture with 20 ng/mL M-CSF and 50 ng/mL RANKL. **d** Representative TRAP staining images. **e** Quantitative analysis of TRAP staining. Scale bar = 200 μm. **f** Typical flow cytometric analysis of CD11b^hi^Ly6C^hi^ and CD11b^−^Ly6C^hi^ cells from bone marrow cells obtained in LPS-treated NLRC3^−/−^ or WT mice. **g** Quantification on the frequency of CD11b^hi^Ly6C^hi^ and CD11b^−^Ly6C^hi^ cells in **c**. Results were presented in a form of mean ± SD. *P < 0.05, **P < 0.01

The above results were based on OVX mice. Thereafter, this study verified how NLRC3 affected mice with lipopolysaccharide (LPS)-induced inflammatory bone loss. LPS (10 mg/kg) was injected into NLRC3-deficient mice or normal mice through the peritoneal cavity. It was illustrated that µCT analysis evidencing that NLRC3^−/−^ mice displayed a lower bone mass than the normal mice (Fig. 5a). Quantification analysis showed decreased BV/TV, Tb. N, Ct. Th, and Tb. Th in NLRC3^−/−^ mice with LPS injection; whereas, trabecular separation increased in NLRC3^−/−^ mice with LPS injection than that of WT mice with LPS injection (*p* < 0.01) (Fig. 5b). After the cell separation from the femoral and tibial bone marrow, the flow cytometry analysis found that TNFα+ Th17 cells increased significantly in NLRC3^−/−^ mice with LPS administration (Fig. 5c). The BMM cells from both groups were exposed to 50 ng/mL RANKL as well as 25 ng/mL M-CSF culture for 120 h. TRAP staining of cells showed more accumulated osteoclasts from NLRC3^−/−^ mice with LPS administration than that from the control group, with a high TRAP-positive rate (Fig. 5d, e). Moreover, flow cytometric analysis showed increased CD11b ^hi^Ly6C^hi^ cells and CD11b^–/l^° Ly6C^hi^ cells in the NLRC3^−/−^ mice with LPS administration (Fig. 5f, g). In brief, NLRC3-deficient mice were more prone to inflammatory bone loss.

### NLRC3 attenuated the activation of Th17 cells depending on NF-kB

**Fig. 6:**
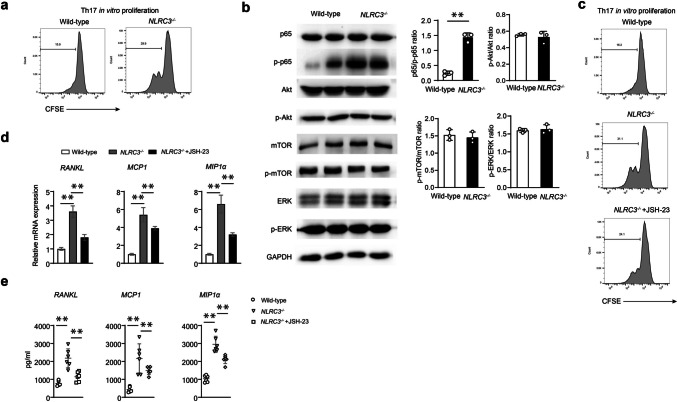
NLRC3 attenuated the activation of Th17 cells depending on NF-kB. **a** The cell proliferation rate of CD4^+^ T cells of each group. **b** Typical WB images and densitometric analysis of p65, p-p65, Akt, p-Akt, mTOR, p-mTOR, ERK, and p-ERK of sorted CD4^+^ cells from wild-type or NLRC3^−/−^ mice. **c** Histograms showing CD4^+^ T cells labeled with CFSE. **d** Quantitative PCR analysis of *RANKL*, *MCP1*, and *MICP1a* of each group. **e** Quantitative ELISA analysis of RANKL, MCP1, and MICP-1α of each group. Data are shown as mean ± SD. *P < 0.05, **P < 0.01

We investigated whether or not NLRC3 regulated Th17 cell proliferation. Hence, CD4^+^ T cells obtained in NLRC3^−/−^ and WT mice were labeled using CFSE (fluorescent dye) and later probed using the anti-CD3/anti-CD28 antibodies in the Th17 (IL-6/TGF-β)-cell polarization conditions to induce cell proliferation for three consecutive days. The cell proliferation rate of NLRC3^−/−^ CD4^+^ T cells is around twice increased relative to WT cells (Fig. 6a). These findings depicted that NLRC3 negatively regulates Th17 cell proliferation.

Furthermore, we initiated to reveal the underlying molecular pathways mediating NLRC3’s effect on Th17 cells during osteoporosis. Therefore, we adopted anti-CD3/anti-CD28 antibodies to stimulate CD4^+^ T cells in wild type and NLRC3^−/−^ mice under Th17 polarization conditions. As a result, NLRC3-deficit within CD4^+^ T cells leads to higher phosphorylation of p65. However, the phosphorylation of extracellular regulated protein kinases (ERK), mechanistic target of rapamycin (mTOR), and serine-threonine kinase (AKT) and did not differ between the two groups (Fig. 6b). Next, we studied the effect of inhibiting NF-kB on Th17 cell response-ability. Results show that the NF-κB inhibitor (JSH-23) retuned the frequency of Th17 cell proliferation by NLRC3-deficiency (Fig. 6c). At the same time, qPCR and cytokine analysis showed that JSH-23 significantly regained the up-regulation of RANKL, TNF-α, MCP-1, and MIP-1α by NLRC3-deficiency in Th17 cells (Fig. 6d, e).

## Discussion

We performed this study to investigate NLRC3’s role in regulating Th17 cell activation as well as osteoclastogenesis during osteoporosis, and the associated molecular mechanisms. The obtained findings have suggested the potential of NLRC3 to attenuate osteoclastogenesis, thus promoting CD4^+^ T cell functions and their ability to recruit CD11bhiLy6Chi inflammatory monocytes into the bone marrow; thereby, restricting osteoporosis progression. Also, NLRC3 negatively affected OC formation through the NK-κB pathway regulation in vitro and in vivo CD4^+^ T cells. Findings from our study have favored NLRC3 as the candidate therapeutic target for managing dysregulation of the adaptive immunity inducing osteoporosis.

Our initial observation revealed that NLRC3 deficiency in OVX mice exacerbated bone loss and osteoclastogenesis. Our study focused on the direct influence of NLRC3 on osteoclast (OC) differentiation, for which we used primary cultured NLRC3^−/−^ BMMs and subjected them to RANKL and M-CSF treatments for OC differentiation. NLRC3 deficiency in BMMs showed no significant effect on OC formation. While considering NLRC3’s effect on the activation and differentiation of T cells, this study tested the bone marrow TNF-α^+^Th17 frequency. Herein, according to our findings, NLRC3 deficiency dramatically elevated bone marrow TNF-α^+^ Th17 cell number [9]. Previous studies have shown TNF-α^+^ Th17 as the main cell causing osteoclastogenesis, supporting our findings. As revealed by evidencing studies, NLRC3 increases many genes related to the activation of Th1/Th17/T cells, such as Ifng, Tnf, Il17f, Il17a, and Tbx21; also, study reveals that Nlrc3 expression in T cells inhibits autoimmunity as well as CD4^+^ T cell responses specific for virus [15].

Increasing evidence is there to suggest that specific T cell subpopulations show direct effect on inducing OC formation through generating some cytokines that cause bone loss in various pathological conditions. Tobias Schmidt et al. demonstrated Th17-induced bone resorption mediating bone loss in primary sclerosing cholangitis [16]. Following this study, Th17 cells producing TNFα are suggested to relate to OC differentiation inducing among inflammatory bowel disease (IBD) patients [9]. Altogether, the evidence indicates that Th17 cells are OC differentiation promoters because they induce the secretion of OC factor RANKL. Hence, our results satisfactorily prove that NLRC3 inhibits the TNF-α and RNAKL expression in Th17.

Since monocytes constitute the OC source, the study shows evidence of the mouse OC derived from the bone marrow CD11b^−/l^ Ly6C^hi^ monocyte progenitor cells and blood CD11b^hi^ Ly6C^hi^ monocytes [17]. In the bone marrow, CD11b^−/l^° Ly6C^hi^ monocyte progenitor cells differentiate more likely than CD11b^+^ MN into OC due to the adverse effects of CD11b and β2-integrin signaling on OC differentiation [18]. Furthermore, studies in vitro reveal that Ly6C^hi^ MNs differentiate efficiently than Ly6C^l^° monocytes into mature OC [5]. Importantly noticeable is the bone marrow CD11b^−/l^° Ly6C^hi^ population that has been significantly expanded in various pathological conditions such as the inflammatory arthritis model. Our observations from this study show that in NLRC3^−/−^ mice, the ratio of CD11b^–/l^° Ly6C^hi^ OC precursors significantly increased because NLRC3 inhibited the inflammatory chemokine secreted by CD4^+^ cells; hence, thereby reducing the recruitment of OC precursor cells to the bone marrow. Furthermore, we found that NLRC3 attenuated the activation of Th17 cells depending on NF-kB pathway in vitro. Meanwhile, we observed that the deficiency of NLRC3 had a major impact on RANKL-induced osteoclast formation. This could be explained by the fact that NLRC3 was not able to activate the NF-κB signaling pathway sufficiently to have a significant effect.

The limitation of transgenic animals did not facilitate us to conditionally knock out the NLRC3 gene in CD4^+^ cells for investigating its effect on Th17 response and bone homeostasis. Furthermore, NLRC may also play a role in osteoporosis via other types of cells, which can be investigated through further high-throughput signal pathway research.

On the whole, this study demonstrated that NLRC3 prevents bone loss by inhibiting TNFα^+^ Th17 cell response without affecting BMM osteoclastogenesis. Also, NLRC3 attenuates TNFα^+^ Th17 cell accumulation into the bone marrow in osteoporosis. The present work revealed NLRC3’s key effect on regulating bone mass in a CD4^+^ cell-dependent manner through adaptive immunity. Altogether, our evidenced results indicate that NLRC3 can be used as a potential target for osteoporosis treatment.

## Methods

### Mice and modeling of ovariectomy-induced osteoporosis

The NLRC3^−/−^ mice (#NM-KO-210309) were purchased from the Shanghai Model Organisms Center, Inc. This study kept the female NLRC3^*−/−*^ or wild-type (WT) C57BL/6 mice in animal house in standardized environment (12 h/12 h light/dark cycles) and were allowed to drink water and eat food. Afterwards, all animals were ovariectomized (OVX) when they were 20 weeks old. In specific stages of every study, euthanasia of OVX mice was carried out for the collection and subsequent ex vivo analysis of the bilateral femurs and tibias. Each experimental procedure gained approval by the Institutional Animal Care and Utilization Committee of Guizhou Provincial People’s Hospital, China.

### RAW isolation and OC formation in vitro

First of all, this study isolated bone marrow macrophages (BMMs) from the femurs and tibias. The red blood cell (RBC) lysis buffer (Beyotime, Shanghai, China) was used to treat bone marrow cells for 5 min and then cultured in alpha-modified minimal essential medium (α-MEM) (HyClone) that contained 10% fetal bovine serum (FBS) (HyClone), 1% mixture of penicillin-streptomycin, and murine macrophage colony-stimulating factor (M-CSF, 30 ng/mL) (R&D Systems). Afterwards, the culture was subjected to overnight incubation within the humid incubator under 37 °C and 5% CO_2_ conditions. After removing medium at 3 days later, without any operation, those cells adhered to the dish were considered as BMMs that could be used for subsequent experiments.

Osteoclast differentiation, using RAW264.7, was stimulated by 30 ng/mL M-CSF and 50 ng/mL RANKL (R&D Systems). Similarly, OC differentiation, using BMMs obtained as described above, was triggered with 30 ng/mL M-CSF and 50 ng/mL RANKL. Tartrate-resistant acid phosphatase (TRAP) was later utilized to stain those adhered cells, among which, the TRAP-positive multinucleated cells, including over 3 nuclei, were quantified using the software ImageJ.

### CD4^+^ T cell activation in vitro assay

We obtained splenic cells in NLRC3^−/−^ and wild-type mice. Next, we negatively selected the CD4^+^ T cells using the mouse CD4^+^ T Cell Isolation Kit (Miltenyi Biotec). Then, we cultivated the above cells into the 96-well round-bottom plates, using 1 mg/mL Ionomycin, 1 mg/mL plate-bound anti-CD3, and 2 mg/mL soluble anti-CD28 or 50 ng/mL soluble PMA, containing complete RPMI1640 medium that contained 10% FBS, 1% penicillin-streptomycin, 55 mM 2-mercaptoethanol, 4 mM L-glutamine, and nonessential amino acids together with 1 mM sodium pyruvate. Furthermore, we performed Th17 differentiation assays in the presence of 20 ng/mL IL-6, 10 mg/mL anti-IFN-γ, 2 ng/mL TGF-β, and 10 mg/mL anti-IL-4.

### Flow cytometry analysis

The cells were harvested and washed surface staining. Using mixtures comprising isotype-matched controls or fluorescence-labeled mAbs, cells were stained for a period of 30 min on ice. Next, the extracted cells were subjected to 6 h of culture using 500 ng/mL ionomycin and 50 ng/mL phorbol 12-myristate 13-acetate with GolgiPlug (BD Pharmingen) for intracellular cytokine detection. After fixation and permeabilization with Transcription Factor Buffer Set, Perm Buffer III, and BD Cytofix/Cytoperm Fixation/Permeabilization Kit (BD), cells were subjected to intracellular staining. The detected transcription factors or intracellular cytokines were detected by the fluorescence antibodies, followed by quantification with the Beckman-Coulter cytometers. Later, we obtained data in a form of labeled cell proportion in the live-cell gate. The FlowJo software (Tree Star) was then utilized for analysis. Isotype-matched controls were applied as reference for gate setting.

## Adoptive transfer of CD4^+^ T cells

After removing splenic samples from NLRC3^−/−^ and naive WT mice, we used L3T4 MicroBeads (Miltenyi Biotec) for the positive selection of CD4^+^ T cells from splenocyte populations. Finally, each female Rag1^−/−^ mouse was given infection of 2.5 × 10^6^ CD4^+^ T cells through the tail vein.

### Micro-CT (µCT) analysis

The tibia specimens obtained from different groups of mice were overnight fixed in 4% paraformaldehyde. Then, µCT (Skyscan1272, Bruker microCT, Kontich, Belgium) was adopted for scanning these specimens, at the scanning resolution and voltage of 8 µm/pixel and 60 kV, respectively. Thereafter, the NRecon v1.6 software (Bioz, Inc., Palo Alto, CA, USA) was employed for image reconstruction, CTAn v1.9 software (Bruker micro-CT) for image analysis, whereas 3D model visualization software CTVol v2.0 (Bruker micro-CT) for image visualization. The area of interest was defined according to the Bruker micro-CT method annotation.

## Histochemistry

After analyzing the specimens by µCT, they were decalcified using the 0.5 M EDTA decalcification solution for a week at room temperature. The specimens were embedded in paraffin. Then, these blocks were processed by a paraffin microtome for up to 4-µm-thick bone sections for TRAP staining. Next, each section was subjected to dewaxing and washing thrice by PBS. We conducted sectioning as per specific protocols (#387A-1KT, Sigma-Aldrich) and then counterstained the sections for 10 s with Methyl Green (#M884, Sigma-Aldrich, St. Louis, MO, USA).

## Western blotting (WB) assay and antibodies

PMSF-containing cell lysis buffer (Beyotime, Shanghai, China) was utilized for cell lysis, and the solution was measured for the concentration. Next, after mixing equal amounts of proteins, the mixture was boiled using the 5× loading buffer (Beyotime, Shanghai, China). We analyzed approximately 30 µg of protein through 10% SDS-PAGE, followed by electroblotting on the PVDF membranes at 330 mA for 130 min. Thereafter, 5% skim milk was utilized to block membranes for 2 h prior to overnight incubation under 4 °C using a specific primary antibody under gentle rotation. Again, a secondary antibody was utilized to incubate membrane for 1.5 h under ambient temperature, with GAPDH being the endogenous reference for WB assay. The obtained immune response bands were observed by the ChemiDoc XRS+ imaging system (Bio-Rad), then analyzed with the ImageJ software. We used the following antibodies from Cell Signaling Technology (CST): Phospho-NF-κB p65 (Ser536) (93H1) Rabbit mAb, NF-κB p65 (C22B4) Rabbit mAb, mTOR (7C10) Rabbit mAb, Phospho-mTOR (Ser2481) Antibody, Akt (pan) (11E7) Rabbit mAb, Phospho-Akt (Thr308) (D25E6) XPRabbit mAb, p44/42 MAPK (Erk1/2) Antibody, and Phospho-p44/42 MAPK (Erk1/2) (Thr202/Tyr204) (20G11) Rabbit mAb.

### Statistical analysis

The unpaired Student’s *t*-tests (two-tailed) were adopted for comparison, whereas one-way ANOVA as well as Bonferroni post hoc test for comparisons among several groups. For all conducted experiments in this study, *P* < 0.05 stood for statistical significance denoted as “*,”, while *P* < 0.01 denoted as “**.”

### Supplementary Information

Below is the link to the electronic supplementary material.

Supplementary file1 (JPG 778 KB)

Supplementary file2 (JPG 504 KB)

## Data Availability

The data that support the findings of this study are available from the corresponding author upon reasonable request.

## Abbreviations

BMMs: Bone marrow-derived macrophages
BSA: Albumin from bovine serum
BV/TV: Bone volume/total tissue volume
DM: Dulbecco’s modified Eagle’s medium
EDTA: Ethylene diaminetetraacetic acid
FBS: Fetal bovine serum
GAPDH: Glyceraldehydes3-phosphatedehydrogenase
H&E: Hematoxylin and eosin
OC: Osteoclast
OP: Osteoporosis
M-CSF: Macrophage-colony stimulating factor
MN: Monocytes
NLRC3: NOD-like receptor family CARD domain containing 3
PBS: Phosphate-buffered saline
RANKL: Receptor activator of nuclear factor-κB ligand
RIPA: Buffer, radio immunoprecipitation assay
TNF: Tumor necrosis factor
TRAP: Tartrate-resistant acid phosphatase
α-MEM: Alpha minimum essential medium
μCT: Micro-computed tomography
