# Supplementary figures and images for: NLRC3 attenuates osteoclastogenesis by limiting TNFα+ Th17 cell response in osteoporosis
Source: J Mol Med (Berl). 2024 Mar 4;102(5):655–65. doi: 10.1007/s00109-024-02422-y (PMC11055730; doi:10.1007/s00109-024-02422-y)

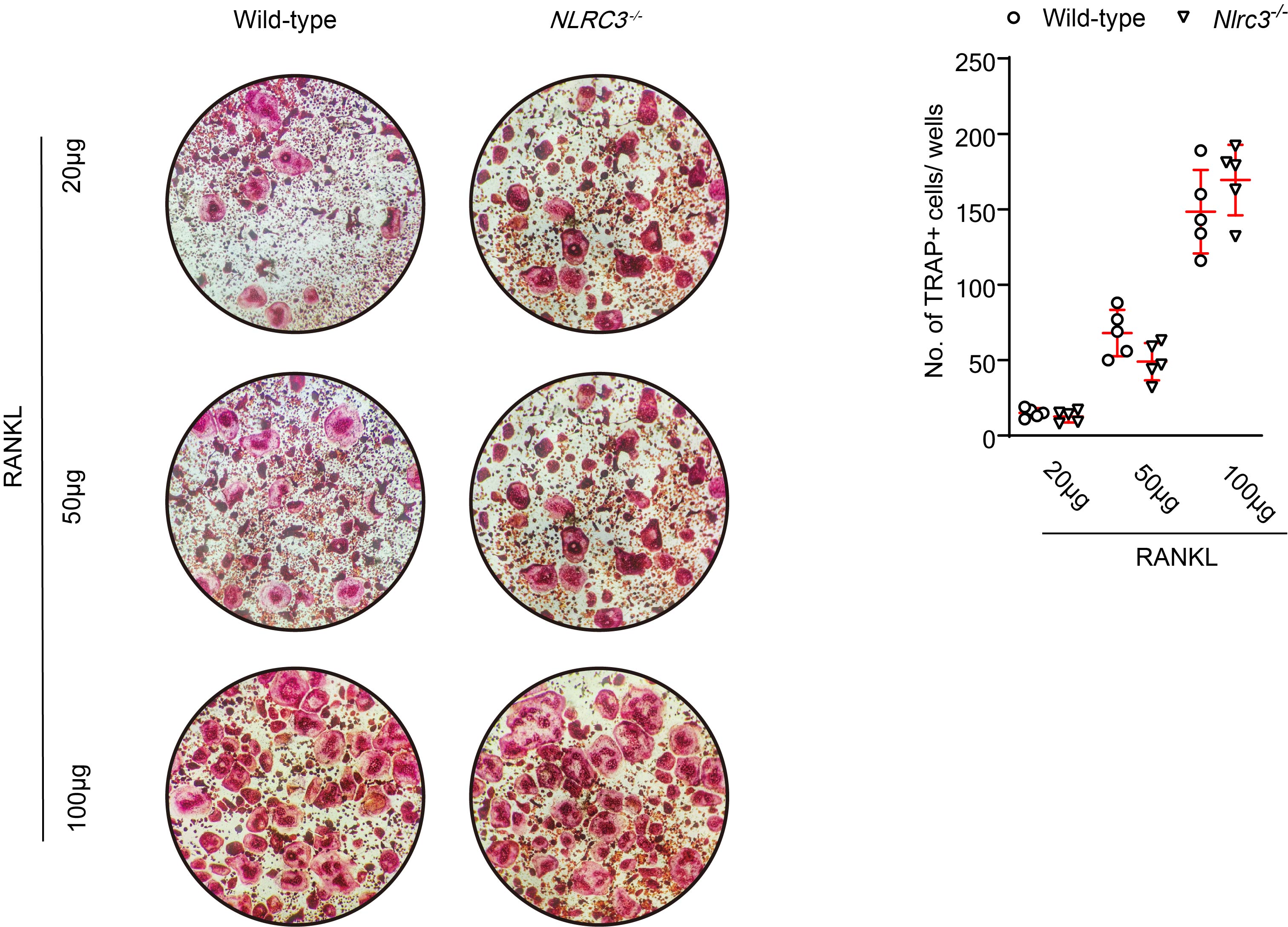

Supplement: Supplementary file 1 — Supplementary file1 (JPG 778 KB) [file 109_2024_2422_MOESM1_ESM.jpg]

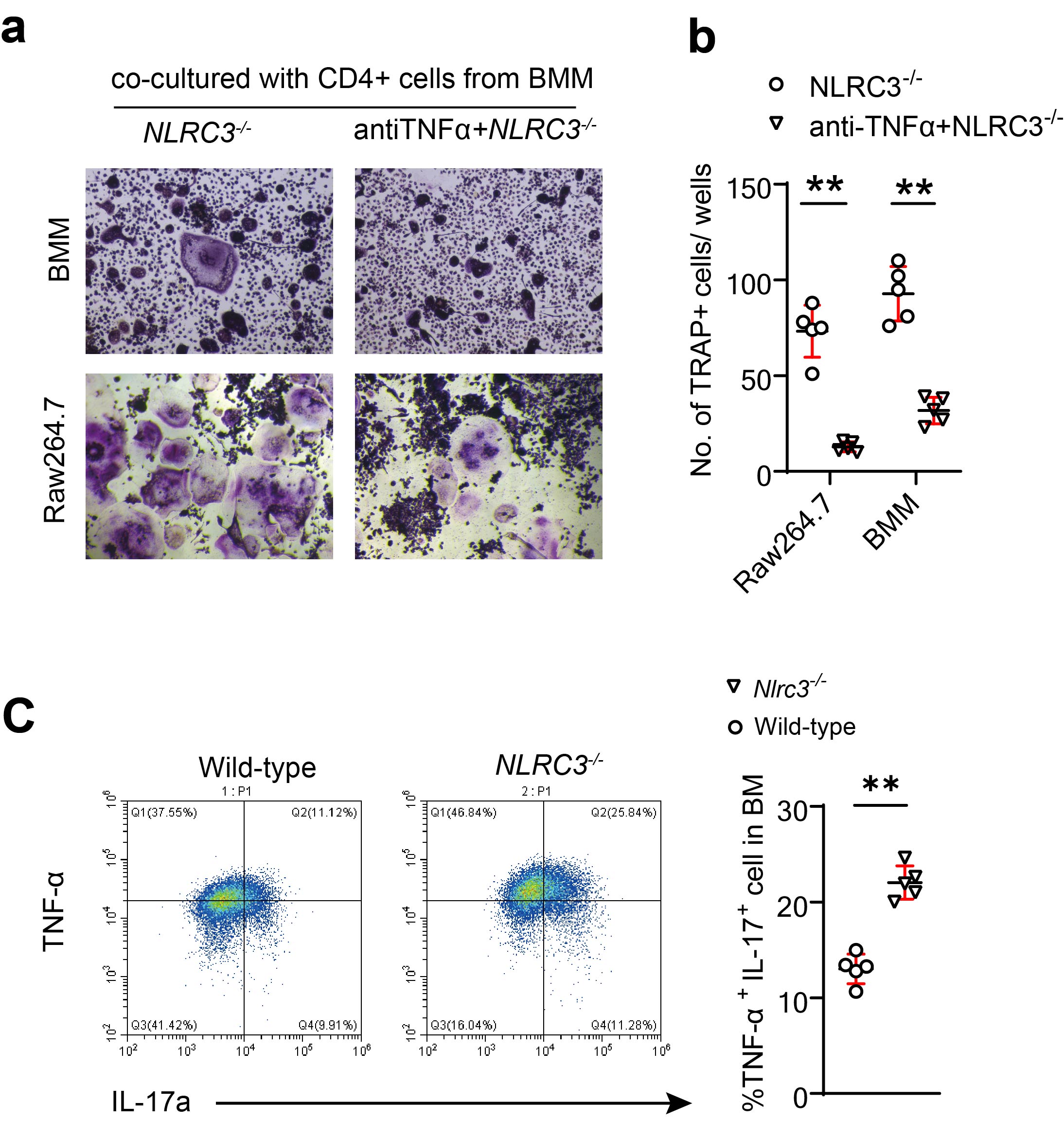

Supplement: Supplementary file 2 — Supplementary file2 (JPG 504 KB) [file 109_2024_2422_MOESM2_ESM.jpg]
